# Medical residents’ perceptions of group biases in medical decision making: a qualitative study

**DOI:** 10.1186/s12909-024-05643-4

**Published:** 2024-06-14

**Authors:** Justin J. Choi, Nada Mhaimeed, Dabia Al-Mohanadi, Mai A. Mahmoud

**Affiliations:** 1 Department of Medicine, Weill Cornell Medicine, New York, NY USA; 2 Weill Cornell Medicine, P.O. Box 24144, Doha, Qatar; 3 Department of Medicine, Johns Hopkins University, Baltimore, MD USA; 4 Hamad Medical Corporation, Doha, Qatar

**Keywords:** Bias, Group decision making, Groupthink, Team dynamics

## Abstract

**Background:**

Systematic biases in group decision making (i.e., group biases) may result in suboptimal decisions and potentially harm patients. It is not well known how impaired group decision making in patient care may affect medical training. This study aimed to explore medical residents’ experiences and perspectives regarding impaired group decision making and the role of group biases in medical decision making.

**Methods:**

This study used a qualitative approach with thematic analysis underpinned by a social constructionist epistemology. Semi-structured interviews of medical residents were conducted at a single internal medicine residency program. Residents were initially asked about their experiences with suboptimal medical decision making as a group or team. Then, questions were targeted to several group biases (groupthink, social loafing, escalation of commitment). Interviews were transcribed and transferred to a qualitative data analysis software. Thematic analysis was conducted to generate major themes within the dataset.

**Results:**

Sixteen interviews with residents revealed five major themes: (1) hierarchical influence on group decision making; (2) group decision making under pressure; (3) post-call challenges in decision making; (4) interactions between teamwork and decision making; and (5) personal and cultural influences in group decision making. Subthemes were also identified for each major theme. Most residents were able to recognize groupthink in their past experiences working with medical teams. Residents perceived social loafing or escalation of commitment as less relevant for medical team decision making.

**Conclusions:**

Our findings provide unique insights into the complexities of group decision making processes in teaching hospitals. Team hierarchy significantly influenced residents’ experiences with group decision making—most group decisions were attributed to consultants or senior team members, while lower ranking team members contributed less and perceived fewer opportunities to engage in group decisions. Other factors such as time constraints on decision making, perceived pressures from other staff members, and challenges associated with post-call days were identified as important barriers to optimal group decision making in patient care. Future studies may build upon these findings to enhance our understanding of medical team decision making and develop strategies to improve group decisions, ultimately leading to higher quality patient care and training.

**Supplementary Information:**

The online version contains supplementary material available at 10.1186/s12909-024-05643-4.

## Background

Medical decision making is an essential component of healthcare and training. It is a complex and dynamic process that is influenced by a wide array of clinical factors (e.g., clinical presentation, disease probabilities, test characteristics, assessment of benefits and harms) and non-clinical factors (e.g., patient preferences and values, characteristics of the clinician and practice setting) [1–4]. An important consideration is that many medical decision making in diagnosis and treatment processes involve the collaboration of multiple individuals—broadly called group decision making [5, 6]. This is especially critical for training skills in medical decision making, which inherently occurs within groups or teams (terms we use interchangeably in this context). This ranges from one-on-one interactions between a trainee and a preceptor to the multifaceted dynamics of a medical team. Yet, the study of group decision making in medical training is relatively underdeveloped.

A common perception of group decision making is that it leads to better decisions by drawing from multiple perspectives, experiences, and areas of expertise. This “wisdom of crowds” has been conceptualized as the collective intelligence that arises when imperfect judgments are aggregated [7]. In medical decision making, collective intelligence has been demonstrated to improve diagnostic accuracy and reduce diagnostic errors by pooling or aggregating multiple physicians’ judgments [8–11]. However, collective intelligence in diagnosis has typically pooled independent judgments without considering the effects of group interactions (or group dynamics).

Certain group dynamics may hinder effective decision making, and some argue that this threatens all group decision making processes [12, 13]. Group biases (or group decision making biases) have been described as systematic patterns of deviation from rational judgment and decision making affecting groups that result in poor decisions [14]. For example, brainstorming in groups may lead to fewer ideas and less productivity [15–18], which might occur due to a group bias known as ‘social loafing’ (when some team members give less effort to problem solving in a group setting) [19]. It is unknown how impaired group decision making in patient care may affect medical trainees’ experiences and whether certain group biases play an important role.

Thus, this qualitative study aimed to explore medical residents’ experiences and perspectives regarding impaired group decision making and the role of group biases in medical decision making. We focus on residents because they are uniquely positioned on the ‘frontline’ of decision making processes on academic medical wards. They work closely with senior doctors or consultants, other health professionals, and patients, and exclusively make decisions in group settings, under supervision and/or in collaboration with others. We formulated the following research questions: What are the lived experiences of medical residents with group decisions that were suboptimal in patients’ care? What are medical residents’ perspectives on group biases in the context of medical decision making? Insights from this study may inform future studies and interventions that improve group decision making processes for patient care and training.

## Methods

### Study design

This qualitative study used a phenomenological method with thematic analysis underpinned by a social constructivist epistemology, which views knowledge (what we understand and know about the world) as co-created through social processes and focuses on how social interactions shape our understanding of the world [20–22]. Individual semi-structured interviews were conducted to facilitate deeper discussion of complex questions and allow participants to share detailed accounts of experiences, interpretations, and perspectives [23]. This study was conducted at Weill Cornell Medicine-Qatar (WCM-Q) and the affiliated Hamad Medical Corporation (HMC). The study was approved by the WCM-Q and HMC Institutional Review Boards (Weill Cornell Medicine-Qatar IRB 22 − 00001 and the Hamad Medical Corporation reliance acknowledgement MRC-01022045).

### Participants

Internal medicine residents at HMC were recruited by e-mail and provided information outlining study details. Enrolled participants were those who voluntarily responded, completed the consent process as per the protocol, and provided signed informed consent. HMC is the main healthcare provider in the state of Qatar, comprising of 12 hospitals that provide all levels of care. HMC is the main affiliate to WCM-Q, the first medical school established in Qatar where many HMC consultants have affiliate faculty appointments. HMC hospitals serve as the main teaching hospitals in Qatar that offer graduate medical education. Clinicians and trainees at HMC represent a diversity of national backgrounds from the Middle East, North Africa, Asia, Europe, and North America.

### Data collection

In-depth, semi-structured interviews in English were conducted over 5 months, from February 24 to June 6, 2023. Interviews were conducted by MAM via video conferencing due to the COVID-19 restrictions. Each interview lasted approximately 30 to 45 min. Audio recordings of all interviews were transcribed and transferred to a qualitative data analysis software (Quirkos, version 2.5.3). We developed an interview guide (see Supplementary Materials) based on a literature review [24] and research team expertise in teams science (JC) and medical education (DA, MAM). We asked about three group biases drawn from Mannion and Thompson (Table 1) that are relevant to patient safety and medical decision making: ‘groupthink’, ‘social loafing’, and ‘escalation of commitment’ [24]. We did not include a fourth group bias identified by Mannion and Thompson known as ‘group polarization’ given similarities in its roots and antecedents with groupthink, and to ensure an in-depth interview could be completed within a reasonable time period.

**Table 1:** Definitions and examples of group biases from Mannion and Thompson

	Definitions	Examples
Groupthink	Occurs when highly cohesive groups with strongly connected members inhibit the expression of individual opinions	Staff members agree with an idea proposed by a senior doctor, who is viewed as a charismatic leader with a national and international reputation for research, despite uncertain evidence for or against the idea, and little time is given to scrutinizing the idea.
Social loafing	Occurs when a group member giving less effort or reducing motivation to achieve a goal when working in a group than when working alone	A multi-ward clinical audit of nursing records finds that one ward has failed to collect audit data due to the senior nurse’s assumption that her three deputies would complete the audit.
Escalation of commitment	Refers to the tendency for individuals or groups to continue to support a course of action despite evidence that it is failing	Continuing a therapy despite a patient’s clinical deterioration rather than considering alternative treatment options or re-considering alternative diagnoses.

As interviews progressed, in-depth questions were also posed, such as “Can you explain in more detail?”, “What were the dynamics in the group that contributed to [that]?”, and “Are there approaches that might avoid [that]?”. At the end of every interview, residents were asked “What else do you think is critical for us to know about group decision making in medicine that we have not covered?”. The data collection ended when sufficiency was reached [25]. 

### Data analysis

Two researchers (JJC, MAM) used thematic analysis for analyzing qualitative data within Quirkos software [26]. First, both researchers familiarized themselves with the data by repeatedly and actively reading all interview transcripts. The researchers took notes on potential data items of interest, questions, and other preliminary ideas in anticipation of the coding process. Then, codes were independently assigned to relevant data segments (words, phrases, sentences) in the first five transcripts. Afterwards, they met to incorporate codes and discuss discrepancies in developing a preliminary coding framework. The researchers used the coding framework on additional transcripts, added new codes as they were identified, and met several times to reach consensus on suggested codes and how these were assigned to data. After ten transcripts, we developed a complete coding framework, which we then consistently applied to all interviews, including those previously coded. This approach ensured that no additional codes were necessary. Then, JJC reviewed all coded and collated data extracts to look for potential themes of broader significance that related back to the original research questions. In doing so, JJC generated an initial set of themes. MAM read all coded data placed within each theme to ensure proper fit. In the final phase, themes were shared and discussed with all authors to prepare a report of the study findings.

### Reflexivity: role of researchers

JJC, DA, and MAM are medical educators and researchers at WCM-Q (DA and MAM) and its home WCM campus in New York City (JJC). JJC has no role with medical residents at HMC. DA and MAM have medical education leadership roles at WCM-Q and HMC; DA primarily interfaces with medical residents as a program director, and MAM primarily interfaces with medical students as a clerkship core faculty. During the conduct of this study, NM was a medical student at WCM-Q.

NM and DA were not involved in data collection or data analysis, and instead, engaged in reviewing themes (from the perspectives of both a trainee and supervisor) to enhance the accuracy and credibility of the research findings. MAM conducted all interviews and maintained an awareness that her role might impact residents’ willingness to share experiences. Throughout their analysis of the dataset, JJC and MAM maintained awareness of potential bias and actively sought evidence of contrary views through reflection and discussion during regular researcher meetings.

## Results

Sixteen medical residents were enrolled and then interviewed, including five first-year (31%), five second-year (31%), and six third-year residents (38%). Eight (50%) were female. Residents recalled instances of suboptimal group decisions for four domains of medical decision making: [1] diagnosis (diagnostic errors); [2] treatment (treatment errors); [3] discharge (premature discharge); [4] monitoring (delayed recognition of clinical deterioration). The influence of the consultant (i.e., attending physician) on the medical team played a ubiquitous role in suboptimal group decision making. Other team members that were mentioned as part of the group decision making process in these cases were other residents on the team, consulting physicians from other specialties, and in some cases, the case manager or social worker (as part of the discharge process) and the patient or family members themselves (as part of shared decision making). Notably, nurses were not mentioned by any resident in their recall of past experiences with suboptimal group decision making.

There were mixed findings in medical residents’ ability to recognize various group biases in medical decision making. Groupthink was the most readily identified by residents. In all cases of groupthink, residents found themselves following the decision or opinion of the consultant. Only two residents had difficulty recalling an experience representative of groupthink.

For social loafing and escalation of commitment biases, few residents could identify these in their experience with patient care teams (three and five residents, respectively). Several residents viewed these group biases as not plausible in medical decision making given individual motivations and team awareness of promoting quality and safety in patient care. Some residents suggested that decreased participation during team rounds might be misconstrued as social loafing, when in fact it reflects varying roles and responsibilities (e.g., early learners leaning on the experience and expertise of senior team members) or a division of labor (individual work versus tasks requiring collaboration). Anecdotes in which social loafing was identified included a resident’s lack of interest in a specific rotation, senior residents ‘sitting back’ at the end of the year, and a consultant with an extremely ‘hands off’ approach with residents.

For most cases in which escalation of commitment was perceived as a contributor to suboptimal group decisions, faulty diagnostic reasoning (e.g., not questioning the accepted diagnosis, failure to consider other possible diagnoses) played a prominent role. For example, a medical team continued pursuing a diagnosis (e.g., tuberculosis workup) and failed to change an accepted diagnosis (e.g., heart failure exacerbation) despite repeated negative testing and worsening of the patient’s condition, respectively. For one resident, escalation of commitment was most noticeable in management plans, sticking with a treatment plan or protocol despite lack of efficacy.

In addressing medical residents’ perspectives on group biases in medical decision making, 5 themes with subthemes were identified (Table 2). We present and discuss these below with representative quotes.

**Table 2:** Major themes and subthemes summarizing medical residents’ experiences with and perspectives on group biases in medical decision making

Theme 1: Hierarchical influence on group decision making
*Subthemes*
• Top-down decision making • Conformity vs. autonomy • Perceived value in judgments and decisions

Theme 2: Group decision making under pressure
*Subthemes*
• Time pressures during work rounds • Pressure to discharge patients

Theme 3: Post-call challenges in decision making
*Subthemes*
• High patient volume • Increased workload • Fatigue in decision making

Theme 4: Interactions between teamwork and decision making
*Subthemes*
• Communication • Cohesion • Mutual support

Theme 5: Personal and cultural influences in group decision making
*Subthemes*
• Individual personalities • Personal experience and confidence • Cultural influences

### Theme 1: hierarchical influence on group decision making

Most residents viewed group decision making in patients’ care as a top-down process that starts with the consultant. Residents felt there are few opportunities to challenge the consultants on decisions they have made, thus withholding their opinions or questions on any given decision.

*“The decision is made by the most senior member of the team, so we cannot do much about it.”* (Resident 5).

Due to the hierarchy in medical decision making, residents perceived a tension between conforming to the group decision and expressing individual thoughts.

*“[Sometimes] you just go with the flow and you try not to disrupt the group with an individual thought.”* (Resident 4).

*“You just go with the flow and do what you’re supposed to do. You’re not even allowed to question [the consultant’s decision]. Because everyone does this, and for your peace of mind, you don’t argue a lot.”* (Resident 5).

Most residents felt that a lower rank in the medical team hierarchy was associated with lower value placed by more senior team members on their judgments and decisions.

*“Because of your title as a ‘PGY-1’, no one takes your ideas seriously…Hierarchy is the main problem.”* (Resident 12).

On the other hand, other residents reported that some consultants did value the input of the lowest ranking team members.

*“Some consultants want to listen. [They are the ones] who treat you as a physician and are willing to learn more from you, and also to let you learn from [them].”* (Resident 11).

### Theme 2: group decision making under pressure

Residents described several external pressures that influenced both the group decision making process and final decisions made as a group that resulted in unfavorable outcomes. For example, time constraints on work rounds was perceived as a limiting factor in having in-depth discussions regarding patient care decisions. Thus, residents felt they had less time to ask questions or challenge consensus thinking or decisions during work rounds.

*“Rounds are busy. For example, you have to attend morning report from 7 to 8 o’clock, you have to see the patients and pre-round, then you have to do the rounds, and after rounds at 10 o’clock you have [another] activity. Your time will not allow you to argue [with a group decision].”* (Resident 2).

There were also external pressures on discharge decisions of medical teams. The pressure felt by residents to discharge patients was often attributed to a cultural norm of promoting hospital throughput, thereby conflicting with thoroughness in patient evaluations.

*“The team wanted to discharge patients very quickly from the hospital… I think there is this concept of discharging patients. We stabilize and discharge without further investigation.”* (Resident 10).

Residents also perceived pressure from case managers in questions directed to the team regarding reasons for prolonged hospitalizations.

*“Also, the case manager in the group asking, ‘Why is this patient in the hospital for so long?’ or ‘What’s the indication [for continued hospitalization]?’ contributes to the push for discharging patients.”* (Resident 7).

### Theme 3: post-call challenges in decision making

Residents reported several aspects of post-call days that posed challenges to optimal group decision making. First, post-call days have the highest patient volume due to the number of admissions during call days. One resident recalled a case in which there was a delayed diagnosis of cellulitis due to a suboptimal evaluation of the patient during a busy post-call day with a high patient volume.

*“It was our post-call day, which is usually very busy. We have to see lots of patients. I think this might have contributed to us not taking a good history from the patient and not examining her very well.”* (Resident 14).

Increased workload, which is related to patient volume, was another aspect of post-call days as it pertained to the high number of tasks that need to be executed and may put the team at risk of missing or delaying recognition of a critical finding.

*“When there is higher workload and no one, not even the senior resident, on our team may be able to catch [everything]. We will be overwhelmed with doing the basic [things] that we need to do.”* (Resident 15).

As a result of higher patient volumes and workload during post-call days, residents identified fatigue as a notable influence on impaired group decision making.

*“You cannot sleep [during call], and thus, you’re not focused in a hangover state.”* (Resident 12).

### Theme 4: interactions between teamwork and decision making

Several aspects of teamwork were described as having an influence on the quality of group decisions in patient care. Residents identified a relationship between effective team communication and quality in group decision making.

*“What I believe is really affecting good [decisions] for the patient is doing multiple recaps within the team, always keeping each other updated…If there is miscommunication and no proper updating about the patient, we really cause team malfunction and poor decisions.”* (Resident 15).

Another aspect of teamwork that was important for residents’ motivation to engage in group decision making was the degree to which team members valued each other and their motivation to work together (i.e., team cohesion).

*“The interpersonal relationships between the healthcare workers are really essential for motivating the residents to do their best… If you’re [in a group] with members who are rude and don’t allow you to learn, then you will not feel as motivated to contribute.”* (Resident 5).

Finally, residents who felt better supported by their team members were more engaged in the group decision making process.

I have worked in teams that support you and even the registrar will be very involved in the [group’s decision]. But sometimes it will be opposite.

(Resident 10)

### Theme 5: personal and cultural influences in group decision making

Residents viewed personality characteristics of team members as determinants for engaging in group decision making. For the resident team member, their tendency towards being extroverted versus introverted was identified as being informative of how engaged they would be in discussions around group decisions. The personality of the consultant was also viewed as important.

*“I once had this very nice group dynamic that was very dependent on the consultant’s personality. He was very nice and let us all [contribute to] discussions on the patient. Because everyone was involved, we could finalize a good decision.”* (Resident 12).

Most residents cited their inexperience or lack of confidence as barriers to speaking up in group decisions driven by more experienced clinicians.

*“As a resident, you think that you don’t have any enough experience and knowledge to argue with the consultant or the senior resident, so sometimes you don’t have anything to add [to the group decision making].”* (Resident 1).

Finally, residents identified cultural background as a factor in team dynamics and group decision. In this diverse residency program, consultants and trainees come from both Eastern and Western cultures. Residents attributed some of group bias and nature of group interactions to the diversity of consultants.

*“Sometimes maybe bias can happen [because] we have different nationalities. For example, there are some consultants [who] are demanding. They want you to be 100% perfect. Maybe this can contribute to the team harmony.“​* (Resident 2).

## Discussion

This study aimed to explore medical residents’ perspectives regarding group decision making in patients’ care, including views on the relevance of certain group biases in medical decision making. Findings in this study provide unique insights into the complexities of group decision making processes in teaching hospitals, which is an underdeveloped area of investigation within medical education. The findings reveal several key points that reflect the challenges that residents face regarding team dynamics and group decision making.

We found that team hierarchy can negatively influence residents’ perceptions of group decision making in a teaching hospital. This certainly adds to well-established concerns regarding team hierarchy as a threat to patient safety. Whereas prior studies have also demonstrated the influence of hierarchical structures on speaking up or challenging authority [27–29], this study found that residents perceived a direct link between team hierarchy and suboptimal team decisions, some of which led to adverse patient outcomes. Voogt et al. interviewed medical residents to explore inhibitors and drivers of speaking up and found that nonhierarchical organizations and supervisors with open attitudes enable speaking up behaviors among residents [29]. Our findings build on this work by demonstrating that hierarchy not only affects the nature of team discussions (i.e., who feels comfortable speaking up), but also impacts group decisions (i.e., who is contributing to decisions about patient care). While some residents reported experiences with consultants who created an environment for open discussions, it was more often the case that residents felt their contributions were undervalued by consultants.

Residents identified personal and cultural factors that influenced group decision making and contributed to consultants’ undervaluing their opinions. For example, residents viewed their personal inexperience or lack of confidence as barriers to their participation in group decision making. Residents also identified culture as an important factor in the team dynamics, particularly the nature of interactions between consultants and trainees. Hierarchical structures and power distances between teachers and learners are greater in Eastern cultures than Western cultures [30]. Being in an Eastern culture such as Qatar, many residents alluded to decision making in medical teams as consultant-centered. Thus, consultants who undervalue residents’ contributions to group decision making may reflect cultural norms of the region and training environment. This might also explain why nurses, social workers, or other non-physician health professionals were never mentioned as contributors to group decision making. Eastern cultures also tend to have exhibit uncertainty avoidance [31], which was identified by one resident as an expectation of certain consultants for residents to be “perfect.”

This study also identified various factors perceived by residents as contributors to suboptimal group decisions. Such factors included time pressures imposed on work rounds, external pressures from other staff members, and factors specifically associated with post-call days such as high patient volume, increased workload, and fatigue. Few studies have examined contributing factors or external forces that negatively influence medical team decision making. Lauffenburger et al. studied the influence of factors contributing to suboptimal prescribing decisions by medical residents on night shifts [32]. They also discovered that time pressures and perceived pressure from other staff contributed to suboptimal decisions by medical residents. Other studies have suggested that these factors are modifiable, and that targeted interventions to alleviate factors such as time pressures and fatigue can improve quality and patient safety. Tsiga et al. conducted an experimental study in the primary care setting that compared ‘time pressure’ versus ‘no time pressure’ groups of general practitioners in simulated clinical scenarios and found that under no time pressure, general practitioners were able to ask more questions indicated by guidelines, conduct more thorough clinical examinations, and provide more advice to patients [33]. A recent meta-analysis also found that resident duty hour restrictions, which aim to mitigate adverse post-call factors such as increased fatigue and workload, are associated with improvement in resident-based outcomes (e.g., emotional exhaustion, well-being) without adversely impacting patient-based outcomes (e.g., length of stay, medical errors) [34]. Thus, these factors identified by residents as contributors to suboptimal group decisions are likely modifiable and serve as potential targets for future interventions to improve group decision making and patient outcomes.

Another important finding in our study was that medical residents were able to identify group biases in past experiences to a varying extent. The recognition of group biases may enable the opportunity for critical reflection in educational practices that can amplify learning among residents [35]. Most residents were able to recognize groupthink in their past experiences with group decision making; however, most had difficulty recognizing social loafing and escalation of commitment. We posit there are several underlying reasons for this. First, residents may lack sufficient education on training on decision making sciences and cognitive biases that could equip them with knowledge and skills to be able to detect them in everyday practice. Second, as several residents pointed out, some group biases may be less applicable in medical decision making compared to other domains. This calls for future research that identifies the kinds of group biases that may emerge within varying contexts and settings.

This study has several limitations. The findings are based on the experiences and perspectives of medical residents from a single medical institution, which may limit the transferability of findings to other settings. In addition, asking residents to speak on past experiences may introduce recall bias or social desirability bias (answering in a manner that would be viewed favorably by others). Finally, de-identification of transcripts to preserve participants’ privacy and confidentiality limited any analysis of certain individual characteristics (such as race, ethnicity, nationality, or gender) and their role in group decision making. Future research should focus on exploring team dynamics in group decision making across diverse settings.

In conclusion, we found that residents perceive a negative hierarchical influence on group decision making, view factors such as time pressures and post-call day challenges as contributors to suboptimal group decisions, and are able to identify group biases in their past experiences that may inform future educational practices. Future research is needed to enhance our understanding of group biases in medical decision making and inform the development of targeted interventions aiming to mitigate group biases and ultimately lead to better patient care and education.

### Electronic supplementary material

Below is the link to the electronic supplementary material.

Supplementary Material 1

Supplementary Material 2

## Data Availability

The datasets used and/or analyzed during the current study are available from the corresponding author on reasonable request.
