# Supplementary material for: Medical residents’ perceptions of group biases in medical decision making: a qualitative study
Source: BMC Med Educ. 2024 Jun 14;24:661. doi: 10.1186/s12909-024-05643-4 (PMC11179270; doi:10.1186/s12909-024-05643-4)
Supplement: Supplementary file 1 — Supplementary Material 1 [file 12909_2024_5643_MOESM1_ESM.pdf]

Weill Cornell Medicine-Qatar

Institutional Review Board
Approval Date: April 12, 2022
Expiration Date: April 11, 2023
APPROVED

Interview Guide

We will conduct semi-structured interviews to solicit medical residents' experiences and views on group biases in team decision making in the context of medical training and patient care. This interview guide was developed by the research team and may be revised following a pilot interview that will assess the effectiveness of the interview guide. Each interview will last approximately 30-45 minutes. Each interview will be conducted over video conferencing due to the COVID-19 pandemic. Each interview will be conducted by the same researcher (NM). Each interview will be audio-recorded. Audio recordings will be transcribed into word documents and coded by the research team. Once transcribed, we will destroy all the audio-recordings and only keep the coded, transcribed data.

We are aware that the interviewees can possibly disclose confidential information during this interview, which is why the participants will be instructed not to disclose information that can easily lead to identifying personnel. If the participant does disclose such information, it will be left of out of the transcript.

1. Introduction<br>(5 minutes)	Thank you for joining us today. My name is _______ and I am a [role at the institution].<br><br>We invited you to participate in this study because you are an internal medicine resident and we would like to talk with you about your experiences with group biases in medical team decision making. Our goal is to learn from your experiences. What we learn today will help us identify issues to improve the quality of patient care and medical education. We will treat your answers as confidential. There are no right or wrong answers to any of the questions. I want to emphasize that this entire interview and your participation is strictly confidential. Nothing that you say will be shared with your program directors, attending faculty, or other trainees. You will not be identified in our transcriptions. We will destroy the recordings after transcription is complete. Do you consent to our recording of this interview? Do you have any questions before we begin?
2. Opening question<br>(10 minutes)	I would like to begin with a very general, open-ended question.<br><br>During your time as a resident, can you recall an episode in which a medical decision was made as a group or team, defined as two or more individuals who collaborate in the decision making process, which in retrospect may not have been the best decision?<br><br>Probing questions:<br>"Can you explain in more detail?"<br>"Was there anything going on in the environment that might have contributed?"<br>"Was there anything going on in your team that might have contributed?"<br>"Were there any characteristics of the team leader that might have contributed?"<br>"Was there anything going on personally that might have contributed?"<br>"Did you or others have concerns that this might not be the right decision?"<br>"If so, did you (or they) verbalize that?"<br>"How did this situation make you feel?"
3. Groupthink<br>(5 minutes)	I am going to talk with you about some categories of group decision making dynamics that have been described in non-clinical settings. The first of these is sometimes called *groupthink*. Groupthink is said to occur when highly cohesive groups with strongly connected members inhibit the expression of individual opinions; in such cases, group harmony may take precedence over effective decision-making. Group members desire for harmony or conformity and override their motivation realistically to propose alternative courses of action.<br><br>Opening question:<br>Can you think of an example or a time when you saw this phenomenon present in a group decision making process? What was the ultimate outcome?<br><br>Probing questions:<br>"What were the dynamics in the group that contributed to groupthink on those occasions?"<br>"Are there approaches that might avoid or minimize groupthink?"<br>"Have there been instances when there were consequences for a group member or the patient?"

Institutional Review Board
Approval Date: April 12, 2022
Expiration Date: April 11, 2023
APPROVED

4. Social loafing<br>(5 minutes)	Another factor that has been described as having an effect on group decision making is referred to as *social loafing*. Social loafing is the phenomenon of a group member giving less effort or reducing motivation to achieve a goal when working in a group than when working alone.<br><br>Opening question:<br>Can you think of an example or a time when you saw this phenomenon present in a group decision making process? What was the ultimate outcome?<br><br>Probing questions:<br>“What were the dynamics in the group that contributed to social loafing on those occasions?"<br>“Are there approaches that might avoid or minimize social loafing?”<br>“Have there been instances when there were consequences for a group member or the patient?”
5. Escalation of commitment<br>(5 minutes)	The final group decision making phenomenon we would like to discuss is *escalation of commitment*. Escalation of commitment refers to the tendency for individuals or groups to continue to support a course of action despite evidence that it is failing.<br><br>Opening question:<br>Can you think of an example or a time when you saw this phenomenon present in a group decision making process? What was the ultimate outcome?<br><br>Probing questions:<br>“What were the dynamics in the group that contributed to escalation of commitment on those occasions?"<br>“Are there approaches that might avoid or minimize escalation of commitment?”<br>“Have there been instances when there were consequences for a group member or the patient?”
6. Review and wrap-up<br>(5 minutes)	Those are all the questions that we wanted to ask. What else do you think is critical for us to know about group decision making in medicine that we have not covered?<br><br>Thank you for your time and thoughtful answers. This interview you will remain confidential and the results will be anonymized. If we need to clarify certain points later, would it be okay for me to follow-up with you?<br><br>Thank you.
