# Supplementary material for: Medical residents’ perceptions of group biases in medical decision making: a qualitative study
Source: BMC Med Educ. 2024 Jun 14;24:661. doi: 10.1186/s12909-024-05643-4 (PMC11179270; doi:10.1186/s12909-024-05643-4)
Supplement: Supplementary file 2 — Supplementary Material 2 [file 12909_2024_5643_MOESM2_ESM.docx]

| **Code** | **Description** | **Representative Quote** |
| --- | --- | --- |
| Post-call | Refers to challenges during post-call shifts (after on-call days) | “*Usually in the post-call day you will have a lot of patients*.” |
| Time pressure | Refers to when time constraints on ward rounds or busy schedules influence team decisions negatively | “*Your time will not allow you to argue [with a group decision]*.” |
| Autonomy | Refers to the degree of independence medical residents have in making decisions without senior oversight | “*It encourages the junior to think more, instead of just doing what they’re told*.” |
| Hierarchy | Refers to the ward team structure from more senior to more junior members that influences decision making | “*Sometimes the opinion is valued more from a senior resident than from a junior resident*.” |
| Seeking help | Refers to the willingness of asking for a second opinion or involving another team member in a decision | “*I think when we make group decisions, this is always better than when the single person makes decisions.*” |
| Individual personality | Refers to when individual personality or character traits are cited as having influence on team decision making processes | “*I think it's even related to the personalities of the team members*.” |
| Experience | Refers to how varying levels of clinical experience among team members influence decisions, such as one’s confidence in contributing to team decisions | “*Yes, it's common, actually, especially in PGY-1 because you think that you don't have any enough experience and knowledge to argue with the consultant or the senior resident, so sometimes you don't have anything to add [to the team’s decision].*” |
| Safety | Refers to when an issue of patient safety is raised as a factor in the team decision making process | “*I would say that if the patient was properly assessed with multiple opinions taken into mind, then maybe the patient wouldn't have ended up in the ICU.*” |
| Consultant | Refers to the attending or supervising senior physician and their influence on team decisions | “*You feel that your opinion is not valid or not valued, and there is no opportunity for contrasting your opinion with the consultant’s opinion*.” |
